# Preparation and evaluation of bilayer-core osmotic pump tablets contained topiramate

**DOI:** 10.1371/journal.pone.0264457

**Published:** 2022-02-25

**Authors:** Wen Lin, Yinke Li, Qiongzhi Shi, Xiangru Liao, Yuan Zeng, Wei Tian, Xiangyang Xie, Hui Liu

**Affiliations:** 1 Department of Clinical Laboratory, Huangshi Love & Health Hospital of Hubei Province, Huangshi, Hubei, China; 2 Department of Pharmacy, General Hospital of Central Theater of the PLA, Wuhan, Hubei, China; ISF College of Pharmacy, Moga, Punjab, India, INDIA

## Abstract

Topiramate (TPM) was an antiepileptic agent commonly used in clinical. Studies showed that an oral preparation of TPM with extended-release manner could bring some benefits for epileptics. In this paper, controlled release push-pull osmotic pump (PPOP) tablets of sparingly water-soluble TPM were successfully prepared. This bi-layer tablet core mainly consisted of sodium chloride as osmotic promoting agent and polyethylene oxide as suspending and pushing agents. The influences of osmotic agents, pushing agents and the compositions of coating membrane on TPM release profiles were evaluated. An optimal formulation of TPM-PPOP was obtained through single-factor experiments. *In vitro* release tests showed that the optimum formulation could release TPM at an approximate zero-order rate up to 8 h. Pharmacokinetic behaviors of TPM-PPOP tablets were evaluated and compared with the immediate release capsules after an oral single dose in beagle dogs. Pharmacokinetics results demonstrated that the TPM-PPOP tablet was able to provide a prolonged release of TPM with longer t_max_ and mean residence time. Lower fluctuations of drug plasma levels could also be achieved with TPM-PPOP tablets. These results suggested that sparely water-soluble drugs as TPM can be designed to PPOP for efficacy and safety use.

## Introduction

Topiramate (TPM; 2,3:4,5-Di-O-isopropylidene-β-D-fructopyranose sulfamate), an antiepileptic agent, is commonly used in clinical as a monotherapy or adjunctive therapy for patients with partial onset seizures, or primary generalized tonic-clonic seizures [1, 2]. In late 2012, TPM was approved by the United States Food and Drug Administration (FDA) in combination with phentermine for weight loss [3].

The conventional dosage form of TPM (Topamax®) is a capsule with immediate release beads in it [2]. Although TPM has a relatively long half-life of 21 h *in vivo*, it has not been prescribed as a single dose, partially due to severe adverse effects that often result with peak plasma levels of the drug when taken in high doses [4]. Instead, Topamax® is usually taken in multiple doses, usually twice per day through oral route [5]. The side effects and poor adherence of immediate release TPM is hindering the epileptics from keeping seizure free. To address this issue, a recent strategy in epilepsy management is to use extended-release formulation of antiepileptic drugs [6]. Therefore, an oral preparation of TPM with extended-release manner may be a good option for epileptics, which may reduce the side effects associated with the fluctuating plasma levels of drug, and is preferably administered in a once-daily regimen to enhance the compliance of patients [3, 7–9]. According to the references [9, 10], TPM is preferably prepared into an 8 h sustained-release dosage form.

In order to achieve a constant plasma level, controlled drug delivery systems such as osmotic pump system can be utilized, which can release drugs at an approximate constant rate up to 24 h [11]. Distinguished by its ability to release drugs independently of the environment media composition and hydrodynamics, osmotic pump has many advantages, such as reducing the risk of adverse reactions, increasing the treatment tolerances of patients, diminishing the food effects and possessing comparable *in vitro*/*in vivo* drug release features [7, 12]. Thereby, in the past several decades, various types of osmotic pump systems have been developed to deliver drugs with different aqueous solubilities [13]. Elementary osmotic pump (EOP) was firstly developed [14] with drugs of suitable water solubility (50–300 mg/ml) [15]. Thereafter, porosity osmotic pump was invented, which was designed to deliver water-soluble drugs without drilling an orifice on its surface as EOP [16].

For poorly water-soluble drugs, they could hardly be dissolved in water and herein could not produce enough osmotic pressure by themselves. To enhance the drug release rate, many effective means were carried out to increase the drug solubility. For example, use sulfobutylether-β-cyclodextrin (SBE)_7m_-β-CD as a solubilizer [17], converts active pharmaceutical ingredients (API) into ionic substance by reacting with or adding alkali/acid [8, 18]. In addition, to assist the drug release, certain water-insoluble drugs were released from EOP tablets in the form of suspensions. For example, polyethylene oxide (PEO) was used as a suspending and osmotic agent to prepare nifedipine monolithic osmotic tablet system [19]. However, if the viscosity inside the system was not proper, drug sedimentation might happen and thus lead to incomplete drug release. To date, the push-pull osmotic pump (PPOP) developed by Theeuwes [20] in 1970s is still the most practical way to prepare osmotic pump system for water-insoluble drugs. PPOP can deliver drugs independently of their solubility, because it can release drugs through the orifice as either a solution or suspension under the hydrodynamic pressure generated by the swelling of the push-layer [21, 22]. Therefore, most of the commercially available osmotic pump products containing water-insoluble drugs are of this kind, such as nifedipine push-pull osmotic pump (Procardia XL®, Pfizer and Adalat®, Bayer) and glipizide push-pull osmotic pump (Glucotrol®, Pfizer) [23].

**Fig 1:**
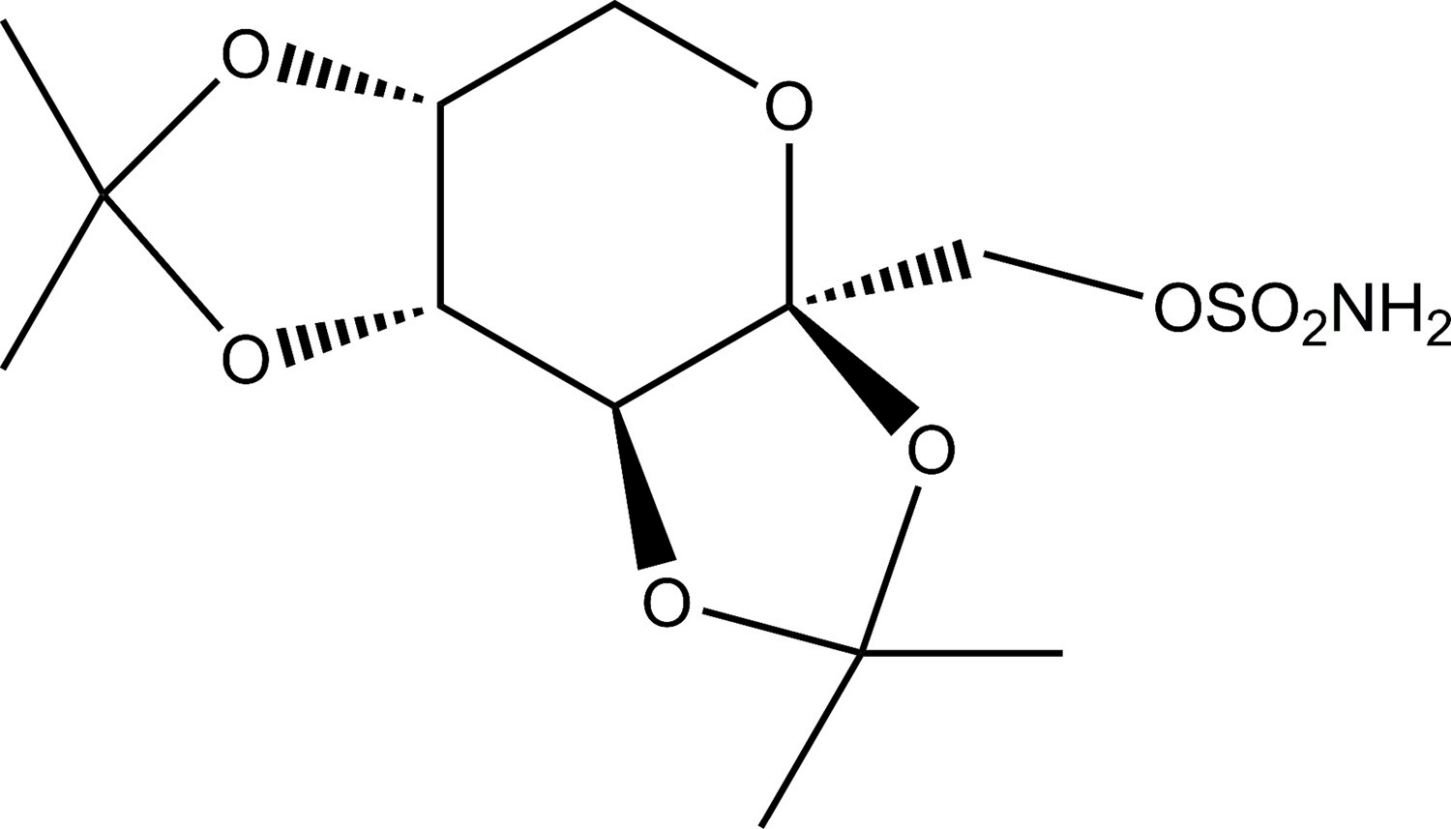
Chemical structural of topiramate.

Besides the conventional capsules, TPM was prepared into the sustained-release pellets as Yang et al. reported [9]. Since TPM (Fig 1) is a sparingly water-soluble drug with solubility in water of 9.8 mg/ml at 23°C [24], it is suitable to be developed into a PPOP system for extended drug release. Although a similar idea for preparing TPM osmotic pump has been used by Alza Company [9], it has not been reported in detail. So far the knowledge of the preparation of TPM-PPOP still remained superficial. The PPOP preparation of TPM will provide an option for the future dosage form development of TPM.

In this work, a PPOP technology was utilized to deliver TPM with a zero-order release profile up to 8 h. Based on single-factor experiments, the influences of PEO molecular weights and levels, NaCl amounts and coating membrane formulations on drug release profiles were investigated to determine the main formulation factors in the PPOP tablets. Orthogonal design was utilized to optimize the formulation with four key factors. Also, the release media and agitation rate on in vitro drug release profile were investigated. Furthermore, the pharmacokinetic profiles of TPM-PPOP tablets were determined, compared with immediate release capsules in beagle dogs.

## Materials and methods

### Materials

TPM was provided by Huayuan Pharmaceutical Co., Ltd (Anhui, China). Polyethylene oxides (PEO) with a molecular weight (Mw) of 100, 200, 300, 4000, 5000 and 7000 kDa (Polyox WSR N-10 NF, WSR N-80 NF, WSR N-750 NF, WSR -301 NF, WSR Coagulant NF and WSR-303 NF) was a gift from Dow Chemical Co. (New Jersey, USA). Magnesium stearate as lubricant was purchased from Huzhou Pharmaceutical Co., Ltd. (Anhui, China). NaCl as osmotic agent was obtained from Nanning Pharmaceutical Co., Ltd. (Nanning, China). Iron oxide red as dyes was purchased from Shanghai iron oxide dyes pharmaceutical Co. (Shanghai, China).

Cellulose acetate (Eastman Chemical Company, Kingsport, TN) with 39.8% acetylene content (M = 30,000 g/mol) was used as a semipermeable membrane. Polyethylene glycol (PEG 400, 1500 and 4000, average molecular weight, analytical grade) was purchased from Yuwang Chemical Reagent Co., (Shandong, China) as plasticizer.

TPM conventional capsules with 25 mg strength (Janssen Pharmaceuticals, Inc., Xian, China) were used as references for comparison. All other chemicals and solvents, which were purchased from Beijing Chemical Agent Co. (Beijing, China), were analytical or high-performance liquid chromatography (HPLC) grade.

### Preparation of TPM-PPOP tablets

The formulations of PPOP tablets investigated were listed in Table 1. Certain amount of TPM, NaCl, PEO and magnesium stearate according to the formulation was passed through an 80-mesh screen respectively and mixed manually to prepare the drug layer. The push layer was obtained by the similar way with appropriate content of PEO, NaCl and magnesium stearate described in the formula. Tiny colorant (Fe_2_O_3_) was added in the push layer for identification. The drug layer and the push layer were compressed into bi-layer tablets by double compression method. The drug layer composition was pre-compressed under 0.5 ± 0.2 kN with single punch press (GY-D10, Shanghai Guoyaolongli Pharmaceutical Device Co., Shanghai, China) and a final compression under a pressure of 6.0 ± 1.0 kN was performed to obtain the tablet with the diameter of 10 mm.

**Table 1:** Core tablet formulations and levels used in the single-factor experiments.

No.	PEO Mws (kDa) ^a^	PEO amount (mg) ^a^	NaCl amount (mg) ^a^	Drug amount (mg)^a^	Magnesium stearate (mg)^a^	PEO Mws (kDa)^b^	PEO amount (mg)^b^	NaCl amount (mg)^b^	Magnesium stearate (mg)^b^	Fe_2_O_3_ (mg) ^b^
F1-F3	100, 200, 300	200	40	50	2	4000	130	35	1	1
F4-F6	100	200, 250, 300	40	50	2	4000	130	35	1	1
F7-F9	100	200	30, 40, 50	50	2	4000	130	35	1	1
F10-F12	100	200	40	30, 50, 70	2	4000	130	35	1	1
F13-F15	100	200	40	50	2	4000, 5000, 7000	130	35	1	1
F16-F18	100	200	40	50	2	7000	100, 130, 160	35	1	1
F19-F21	100	200	40	50	2	7000	130	20, 35, 50	1	1
F22-F24	100	200	40	50	2	7000	130	35	1	1
F25-F27	100	200	40	50	2	7000	130	35	1	1
F28-F30	100	200	40	50	2	7000	130	35	1	1

a: Drug layer

b: Push layer.

The tablet cores were coated in a pan coater (Jiangsu Taizhou Pharmaceutical Device Co., Ltd., China). Cellulose acetate (4.5%, w/v) in acetone/water 95:5 (w/w) containing polyethylene glycol (PEG) was prepared as coating solution. The formulations of coating solution investigated were listed in Table 2. The diameter of the coating pan was 230 mm; pan-rotating rate was 30 rpm; spray rate was 2.5 mL/min; the temperature of inlet air was 50 ± 2°C. After the coating process, the coated tablets were dried at 55°C for 4 h and stored in a container after drying. A 0.60 mm orifice for the release of TPM was drilled on the drug layer membrane face (white face) of the coated tablet by a laser-beam drilling machine (Nanjing Rich Electronic Engineering Technology Industry Co., Ltd., China).

**Table 2:** Coating membrane formulations and levels used in the single-factor experiments.

No.	PEG Mw (kDa)	PEG amount (%,w/v)	Weight gain (%)
F1-F3	1500	1.6	10
F4-F6	1500	1.6	10
F7-F9	1500	1.6	10
F10-F12	1500	1.6	10
F13-F15	1500	1.6	10
F16-F18	1500	1.6	10
F19-F21	1500	1.6	10
F22-F24	400, 1500, 4000	1.6	10
F25-F27	400	0.8, 1.6, 2.4	10
F28-F30	400	1.6	7, 10, 13

For further formulation optimization, single-factor experiments were carried out to screen the main formulation factors that affecting the drug release [10–12, 19] greatly. Before the conducts of the single-factor experiment, formulation factors (e.g. PEO type, PEO amount, NaCl amount) with three levels were arranged in Tables 1 and 2. In each single-factor experiment, the investigated factor (other formulation factors were fixed) with its three levels was conducted, and each factor level had been replicated three times. Then the drug release results were analyzed by similar factor *f*_2_. When the value of *f*_2_ between two formulations was less than 50, the release behaviors of the two formulations were considered statistically different. Thus the investigated factor among the two formulations was recognized as the main formulation factor.

### Quality control and testing

During the preparation of TPM-PPOP tablets, quality control and testing operations as described below were carried out to assure the quality of prepared tablets. Only the prepared tablets fitted the following standards could be tested further.

Water content of the powder: place about 5 g of the powder (from drug layer and push layer, respectively), accurately weighed, in a tared evaporating dish. Dry at 105°C for 1 h, and weigh. The weight loss should not more than 2%.

Content assay of the powder from drug layer: select the final powder of drug layer, equivalent to about 50 mg of TPM, and transfer the powder to a 250 mL volumetric flask containing 130 mL of water; add methanol to volume, and stir for 30 minutes. Centrifuge the resulting suspension to obtain a clear supernatant stock solution. Transfer 5.0 mL of the stock solution to a 25 mL volumetric flask, dilute with mobile phase to volume, mix, and filter to assay in a Waters HPLC system equipped with a Waters Model 410 differential refractometer, a Waters Model 600 pump and a Waters Model 2707 auto-sampler under the following conditions: Vensil XBP C_8_ column (5 μm, 4.6 mm × 150 mm, Agela Technologies); column temperature, 35°C; mobile phase, methanol-water (50/50, v/v); flow rate, 1.5 mL/min; injection volume, 200 μL. The drug content was expressed as % of theoretical formulation content, and the acceptance value should between 98%-102%.

Weight variation: accurately weigh 20 tablets individually, expressed as % of mean tablet weight, of each tablet from the weight of the individual tablet. The acceptance value of each tablet should between 97%-103%.

Content uniformity: accurately measure 10 tablets individually, expressed as % of theoretical tablet content (50 mg), of each tablet from the drug content of the individual tablet. The drug content was assayed by an HPLC as described above. The acceptance value of each tablet should between 92%-108%.

### In vitro release study

*In vitro* release test was performed according to USP paddle method (37°C, 50 rpm, n = 6) using a dissolution apparatus (D-800LS, Precise Apparatus of Tianjin University Co., Ltd, Tianjin, China) in 750 mL phosphate buffer with pH 6.8 [9]. At pre-determined time intervals (1, 2, 4, 6, 8 and 10 h), 5 mL samples were withdrawn, filtered through a 0.45 μm filter, and analyzed by a Waters HPLC system equipped with a Waters Model 410 differential refractometer, a Waters Model 600 pump and a Waters Model 2707 auto-sampler under the following conditions: Vensil XBP C_8_ column (5 μm, 4.6 mm × 150 mm, Agela Technologies); column temperature, 35°C; mobile phase, methanol-water (50/50, v/v); flow rate, 1.5 mL/min; injection volume, 200 μL. The same volume of fresh medium was replaced after sampling.

### Ethics statement

This study did not involve non-human primates. All experiments described in this study were performed in full accordance with the guidelines for animal experiments released by the National Institute of Animal Health. This study is approved by the Animal Care and Use Ethics Committee of the Love & Health Hospital (ETHICS CODE Permit NO.SCXK (Huang) 2018–007). All animals were handled according to the code of ethics in research, training and testing of drugs as laid down by the Animal Care and Use Ethics Committee of the Love & Health Hospital.

### In vivo pharmacokinetic study

Dogs have free access to water (municipal tap water) and food (Navarch Dog Diet). The animals were maintained in the animal facility under controlled environments (12 h light/12 h dark cycle; temperature, 22 ± 5°C; relative humidity, 55 ± 15%). Every effort was made to minimize unnecessary pain and suffering of all animals in the study.

No restraining devices were used in blood collecting procedures. Dogs were restrained gently by trained technicians with the dogs foreleg extended for sample collection of blood from saphenous or dorsal pedal veins. Anesthesia was not used during the blood collecting as this procedure was minimal pain.

No animals were required to be euthanized during this study due to their health status. All animals were healthy, and were released back to the colony when the study was completed.

Six healthy beagle dogs, 3 male and 3 female, having mean weight of 10.4 ± 0.5 kg, were used in a randomized cross-over study with a 2 × 2 Latin square design. The dogs were fasted but had free access to water overnight. Dogs were orally administrated with immediate release TPM capsules (Janssen Pharmaceuticals, Inc., Xian, China) or TPM-PPOP tablets at a dose of 50 mg. Blood samples were collected at 0, 0.5, 1, 2, 3, 4, 6, 8, 10, 12, 24, 36, 48 and 72 h after administration. The obtained plasma samples were stored at -20°C until analysis. A 2-week washout period was provided between each treatment.

The plasma samples were prepared and analyzed according to Mula et al. reported with some modification [4]. Nimesulide aqueous solution (250 ng/mL, w/v) of 40 μL as internal standard and 500 μL of methanol was added to 0.1 mL of plasma. The mixture was vortex mixed for 5 min and centrifuged at 10000 rpm for 10 min (240A, Feige Instrument Company, China), and 20 μL of supernatant was injected into an Agilent 1200 HPLC-MS/MS (Pump G1312B, UV detector G1316B, Autosampler 1367C, Degasser G1379B, G6410A triple quad mass spectrometer, Agilent Technologies, Santa Clara, CA) for analysis. The separation quantification of TPM and nimesulide (internal standard) was achieved by a reversed phase analytical column (Agilent Zorbax C_8_, 5μm, 4.6 mm × 150 mm, Agilent Technologies, Santa Clara, CA) under isocratic conditions, which contained methanol and 0.4 mM ammonium acetate aqueous solution (75:25, v/v) at a flow rate of 0.5 mL/min. A quadratic, 1/concentration weighted, least squares regression algorithm was used to plot the peak area ratio of TPM to its internal standard versus concentration. The lower limit of quantification was the lowest nonzero concentration level, which quantified with acceptable accuracy and precision.

The quantification range of the LC-MS/MS assay was 2–1000 ng/mL. The inter-assay precision was in the range of 9.3 and 11.2% at the quantification range, and the interassay accuracy throughout the quantification range was between -4.08 and -1.23%.

### Statistical analysis

The similarity of dissolution profiles was analyzed using the similarity factor [25], *f*_2_ was defined in the following equation:

$$f_{2}=50\times \log\{{\left[1+\frac{1}{n}\sum {\left(R_{t}-T_{t}\right)}^{2}\right]}^{-0.5}\times 100\}$$

where n was the sample number, Rt the reference assay and Tt the test assay at time point t. If the similar factor (*f*_2_) is not less than 50, the two drug release profiles are considered to be similar.

DAS 2.0 software (Mathematical Pharmacology Professional Communities of China, Shanghai, China) was used on pharmacokinetic data analysis. The significance of difference between the pharmacokinetic parameters was analyzed by using Student’s t-test after the data were log-transformed. All testing was done using the SPSS 13.0 (SPSS Inc., Somers, NY). The level of significance was defined as p value < 0.05.

## Results and discussion

### Influence of PEO molecular weights in drug layer

**Fig 2:**
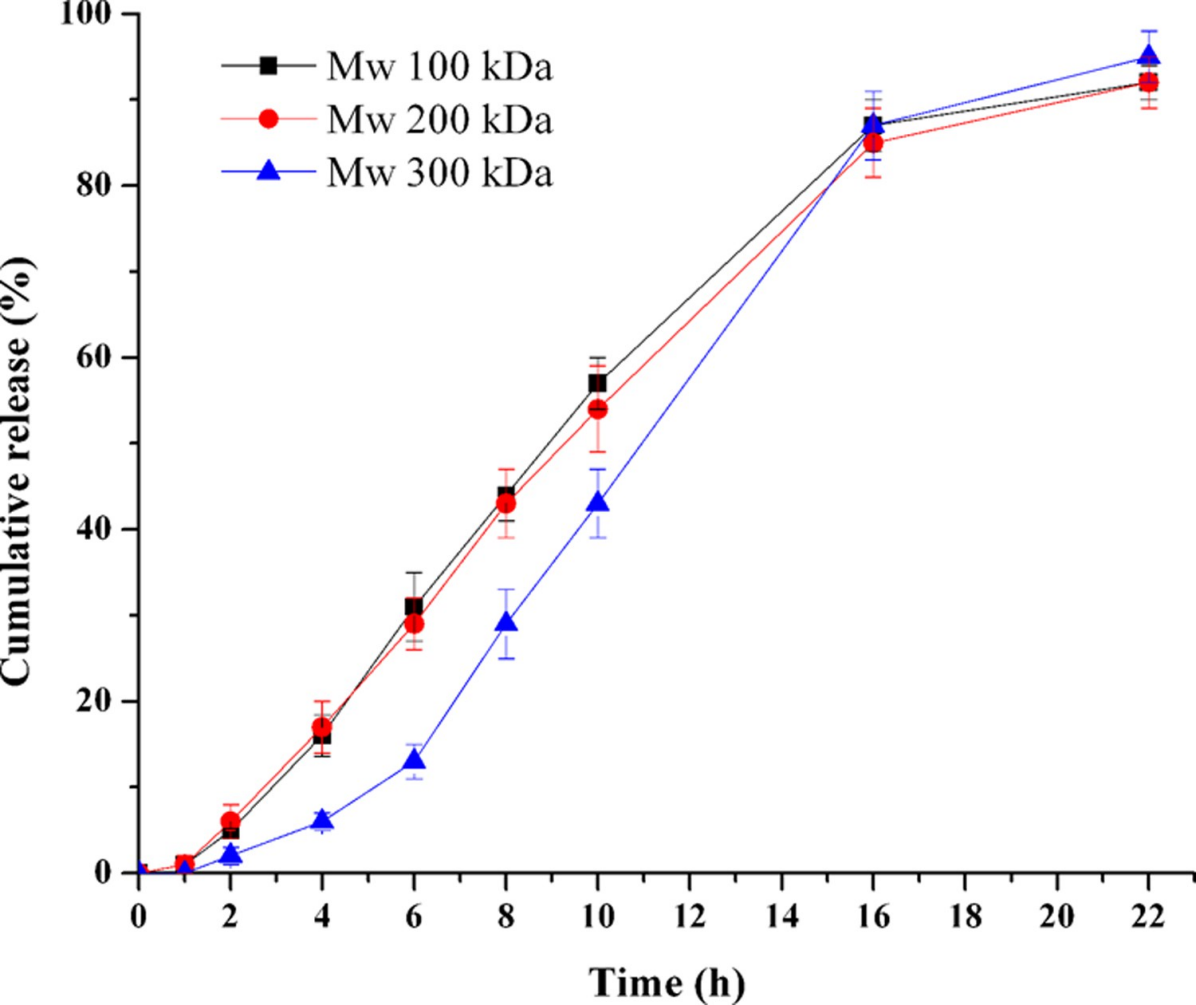
Effect of PEO molecular weight in drug layer on the TPM release (n = 6).

In order to study the influence of PEO molecular weight (Mw) in drug layer on the drug release profile, PEOs with various Mws (from 100 kDa to 300 kDa, F1-F3) were investigated. The release profiles were plotted as shown in Fig 2. Similarity analysis results showed that the 100 and 200 kDa PEOs had a similar *in vitro* release profile (the *f*_2_ between 100 kDa and 200 kDa: 84.1): after about 2 h (lag time) of almost no drug release, the onset of drug release took place at a linear profile until 16 h, after that the TPM release slowed down. The release profile of 300 kDa PEO was different from the other two (the *f*_2_ between 300 kDa and 100 kDa was 46.1; the *f*_2_ between 300 kDa and 200 kDa was 48.2) because the two *f*_2_ values of 300 kDa formulation were smaller than 50. The lag time of 300 kDa PEO around 4 h was longer than the other two, and the drug release rate was greater than the others during the period of 4–16 h, after that the TPM release almost stopped. Thus, the 300 kDa PEO was unfitted to be added in the drug layer for its longer lag time. Considering the tablets with 200 kDa PEO would swell remarkably in the final period of the release test, 100 kDa PEO was selected for the following study.

PEOs with low Mw (100 kDa ≤ Mw ≤ 600 kDa) were usually used in the drug layers to obtain the zero-order release profiles [26]. It was reported that low Mw PEOs hydrated fast in the first 3–4 h and rarely absorbed water afterwards [27]. Therefore, these types of PEOs would imbibe water rapidly, and then formed the flowable drug suspensions in the drug layer. Since PEO with high Mw in push layer could expanded at a constant rate [28], the suspensions in drug layer would be pushed out constantly, and thus a zero order release profile would be obtained.

### Influence of PEO amount in drug layer

**Fig 3:**
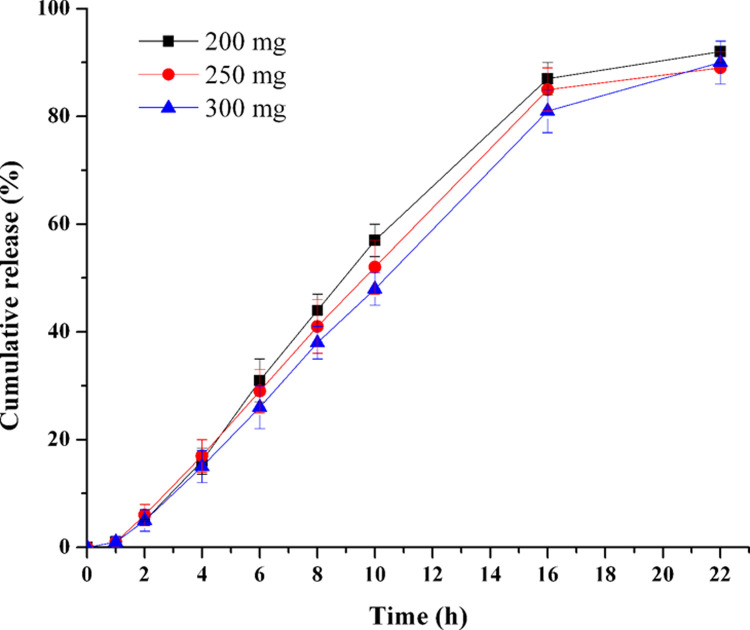
Effect of PEO amount in drug layer on the TPM release (n = 6).

Tablet cores with various PEO levels in drug layer (from 200 mg to 300 mg, F4-F6) were prepared to investigate the effects of PEO amount in the drug layer. As shown in Fig 3, it was seemed that the TPM release decreased as the PEO amount increased from 200 mg to 300 mg, but the difference among them has no statistical meaning (*f*_2_: 200–250 mg: 77.0; 200–300 mg: 62.8; 250–300 mg: 74.8). In order to save the amount of PEO used in the tablets, PEO level was fixed at 200 mg in the following studies.

The augment of PEO amount in drug layer increased the viscosity of drug suspensions, and herein it might increase the stability and dispersibility of TPM in suspensions, which would aid the TPM release [29]. On the other hand, as the PEO levels increase, the viscosity of drug suspensions formed in the drug layer might increase as well, consequently the resist force produced by the viscosity that hindering the fluid out from the orifice will also increase. The positive and negative effects of PEO (within the range of 200 to 300 mg) in the drug layer resulted in no statistical release change finally.

### Influence of NaCl amount in drug layer

**Fig 4:**
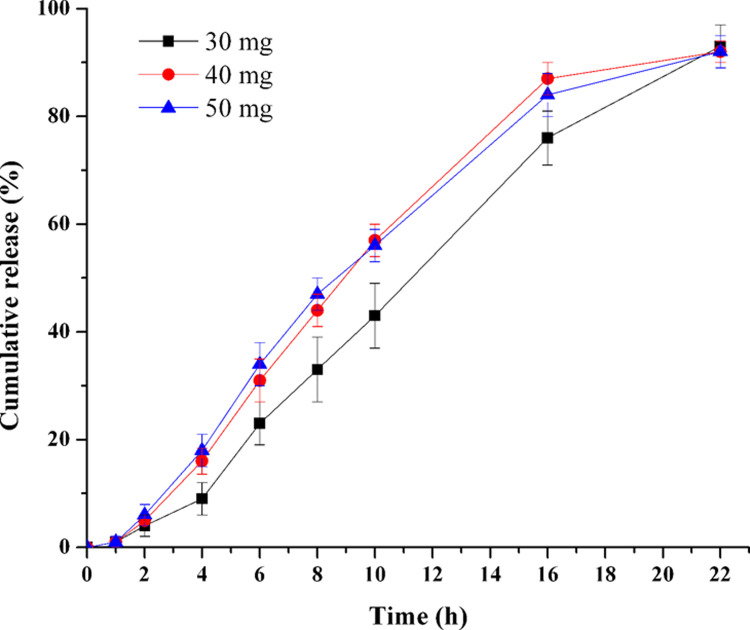
Effect of NaCl amount in the drug layer on TPM release (n = 6).

To investigate the effect of the amount of NaCl on the release of TPM, tablets with various quantities of NaCl (F7-F9) were prepared, respectively. Fig 4 showed that the release increased as the NaCl amount ranged from 30 mg to 40 mg (*f*_2_: 30–40 mg: 48.8), while the change observed between 40 mg and 50 mg NaCl was not remarkable (*f*_2_: 40–50 mg: 78.3). Since more NaCl would bring more osmotic pressure in the drug layer [21, 26], the NaCl in drug layer was positive for the drug release. When the quantity of NaCl extended to 40 mg, the positive effect of NaCl on drug release might reach its maximum. Thus, 40 mg was adopted in the following studies.

### Influence of drug loading

**Fig 5:**
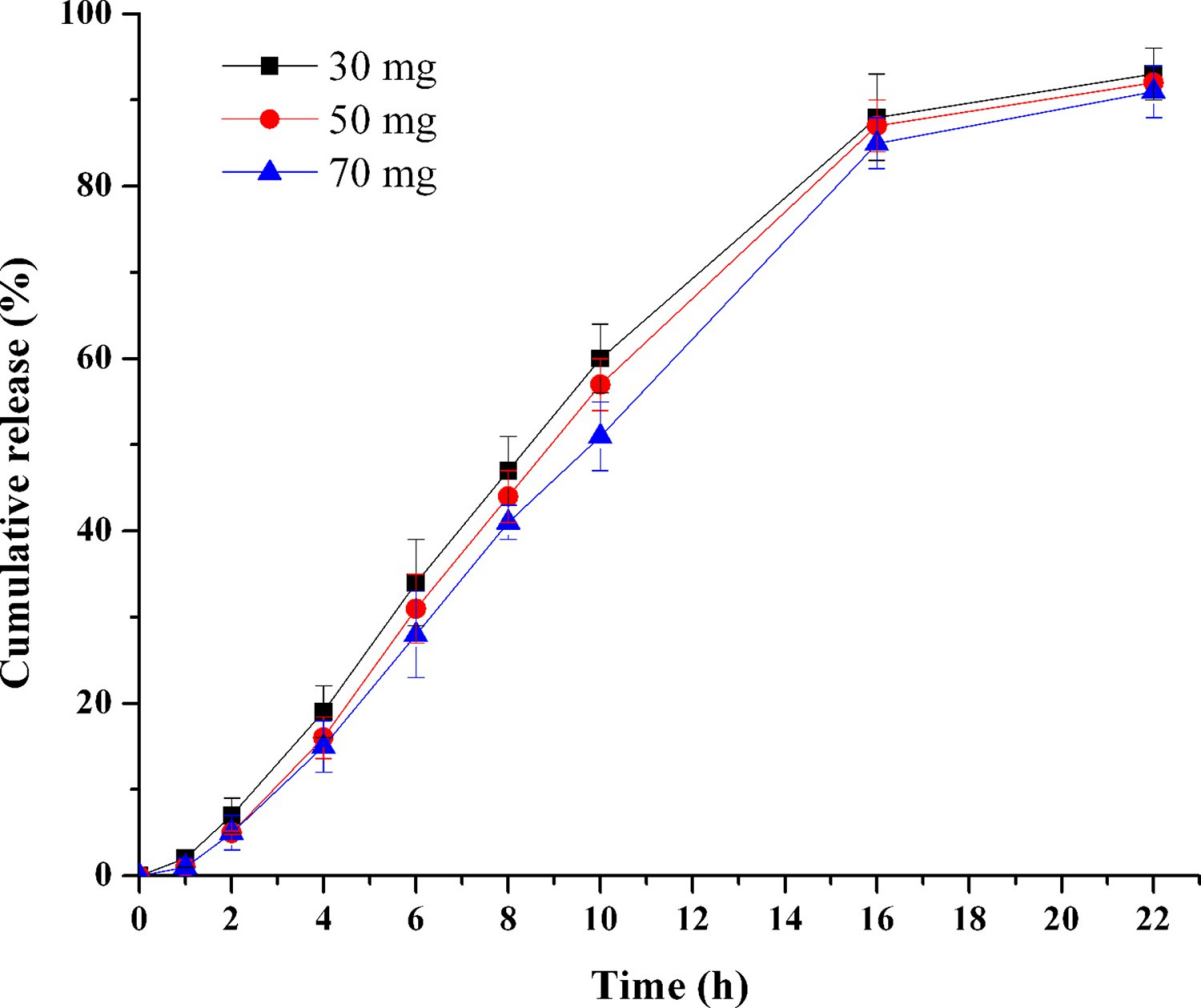
Effect of drug loading on TPM release (n = 6).

The influence of TPM loading was investigated by changing the strength of the tablets (F10-F12). Fig 5 showed the release profiles of various TPM loadings of 30, 50 and 70 mg, and the *f*_2_ of each formulation pairs was 77.6 (30–50 mg), 74.1 (50–70 mg), and 62.6 (30–70 mg), respectively. It revealed that there was no significant difference of release behavior in the tested formulations, when the drug loadings were in the investigated range. It seems that the drug delivery from a PPOP could be considered as independent of the drug loading, because a stable viscous suspension would be formed in the drug layer during the whole release process [22]. In addition, it implied that the drug loading had little influence on the rheology character of drug suspensions. The wide range of the drug loading will provide convenience for future dosage alteration.

### Influence of PEO molecular weights in push layer

**Fig 6:**
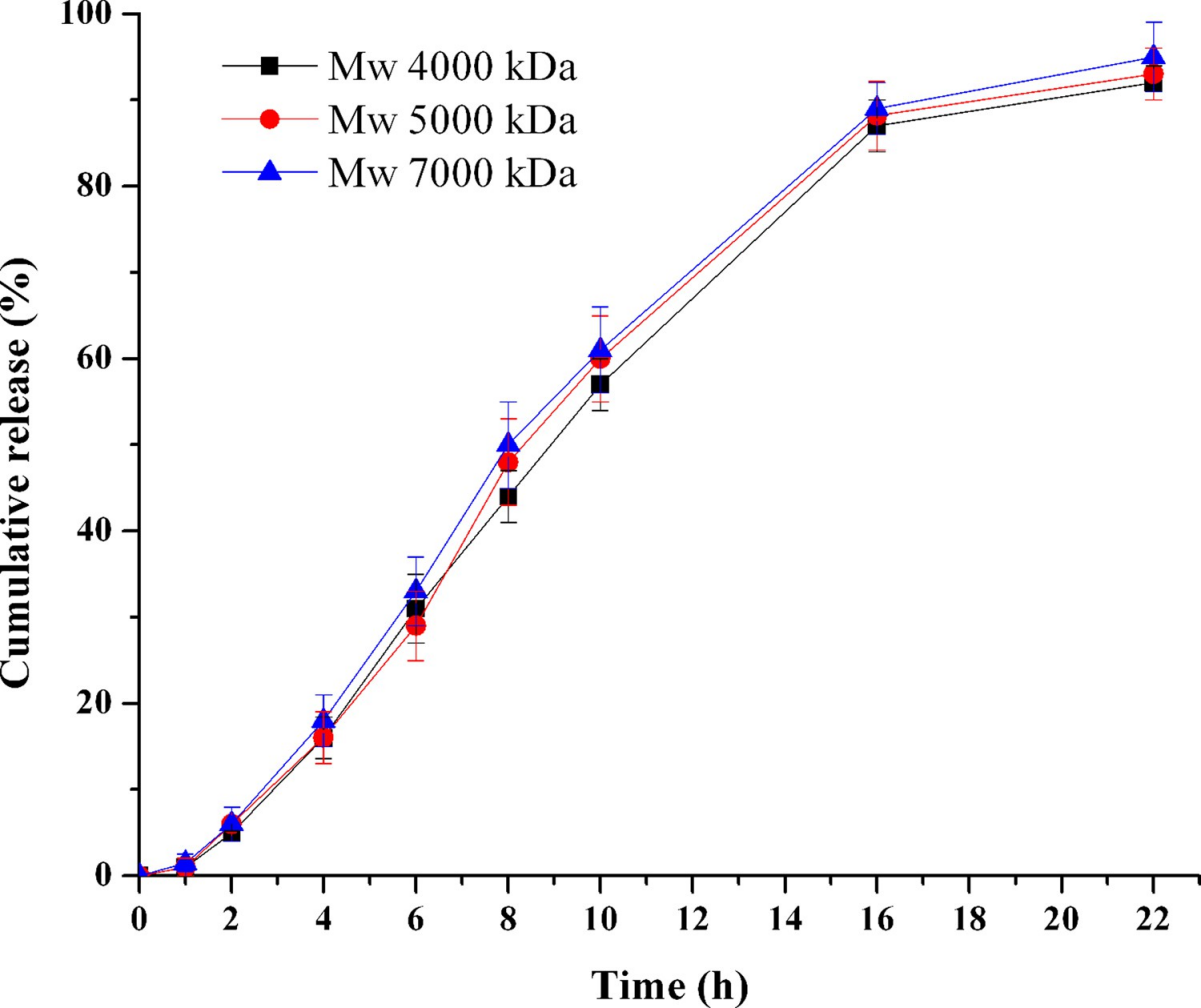
Effect of PEO molecular weight in push layer on TPM release (n = 6).

To investigate the influence of PEO Mws in push layer on drug release profiles, various Mws of PEO (from 4000 kDa to 7000 kDa, F13-F15) were investigated. As shown in Fig 6, the release rate increased slightly as the Mws of PEO increased, the higher Mw PEO was, the more complete drug release. PEO can absorb water and then expand. And the higher Mw of PEO will have a larger expansion volume [28]. Thereby higher Mw PEO has larger expansion volume can push the drug suspensions out of the tablet more complete. Compared with each other in the three release profiles, the *f*_2_ was 85.8 (4000–5000 kDa), 80.3 (5000–7000 kDa) and 75.9 (4000–7000 kDa), respectively. It indicated that the release profiles of the three formulations were not statistically different. However, to obtain a more complete drug release, the highest Mw PEO (7000 kDa) was chosen for the subsequent formulation exploration.

### Influence of PEO amount in push layer

**Fig 7:**
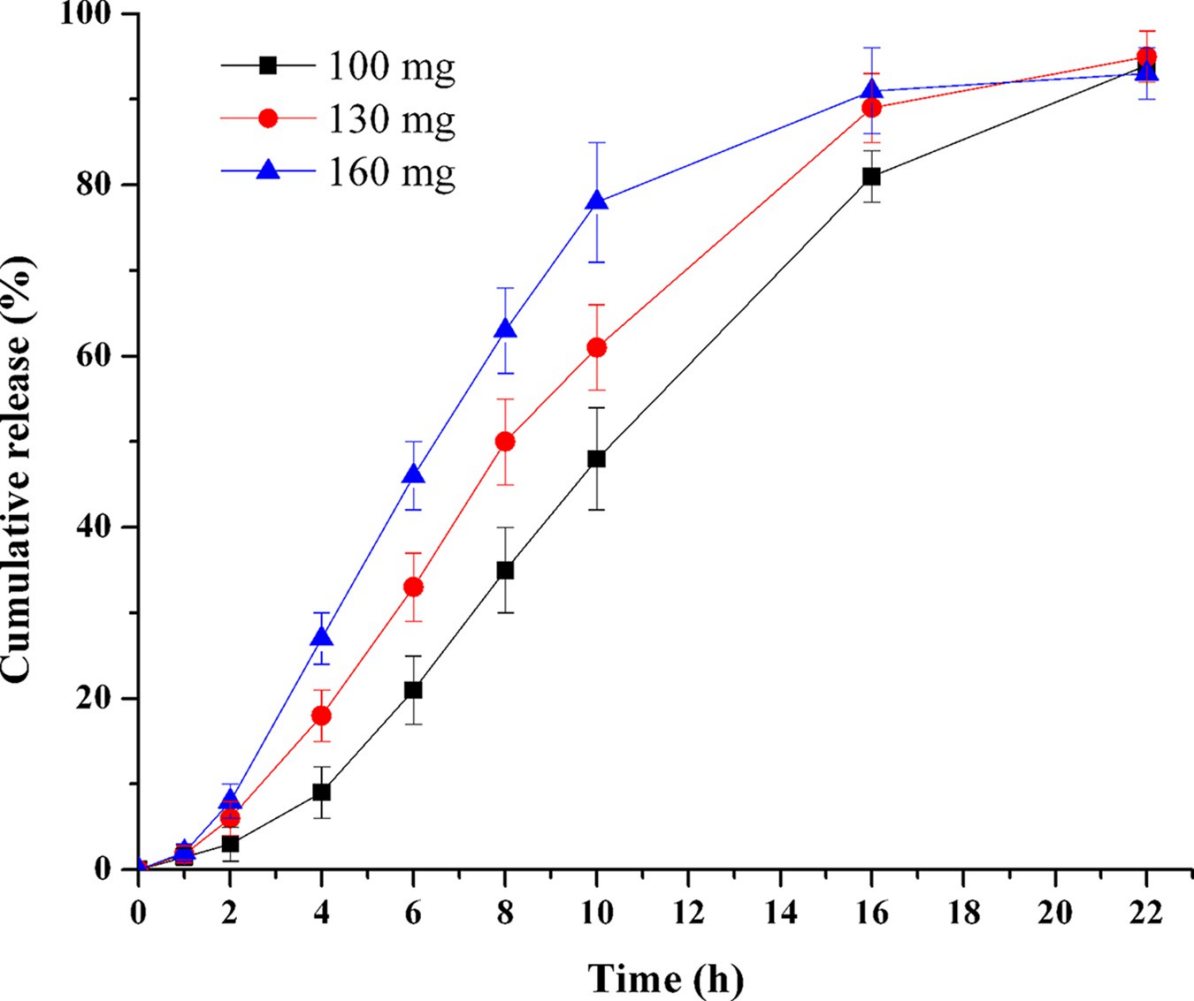
Effect of PEO amount in push layer on TPM release (n = 6).

Various levels of PEO were incorporated in push layer to study the compositive influence of PEO on TPM release (F16-F18). As Fig 7 displayed, the drug release rate increased remarkably as the amount of PEO in the drug layer increased. Compared with each other, the *f*_2_ of the three compared pairs were all smaller than 50 (*f*_2_: 100–130 mg: 48.4; 130–160 mg: 48.0; 100–160 mg: 33.4), which meant the release curves of the three formulations (F16-F18) were significantly different from each other due to the definition of *f*_2_. Thus, this indicated that the PEO amounts in the push layer influenced drug release significantly, and could not be neglected. However, it should be notified that when there was too much PEO in the push layer (e.g. 310 mg) the coating membrane would be deformed. This was because the excess PEO suspensions could not be discharged from the tablet in time and therefore induced the deformation of coating membrane.

### Influence of NaCl amount in push layer

**Fig 8:**
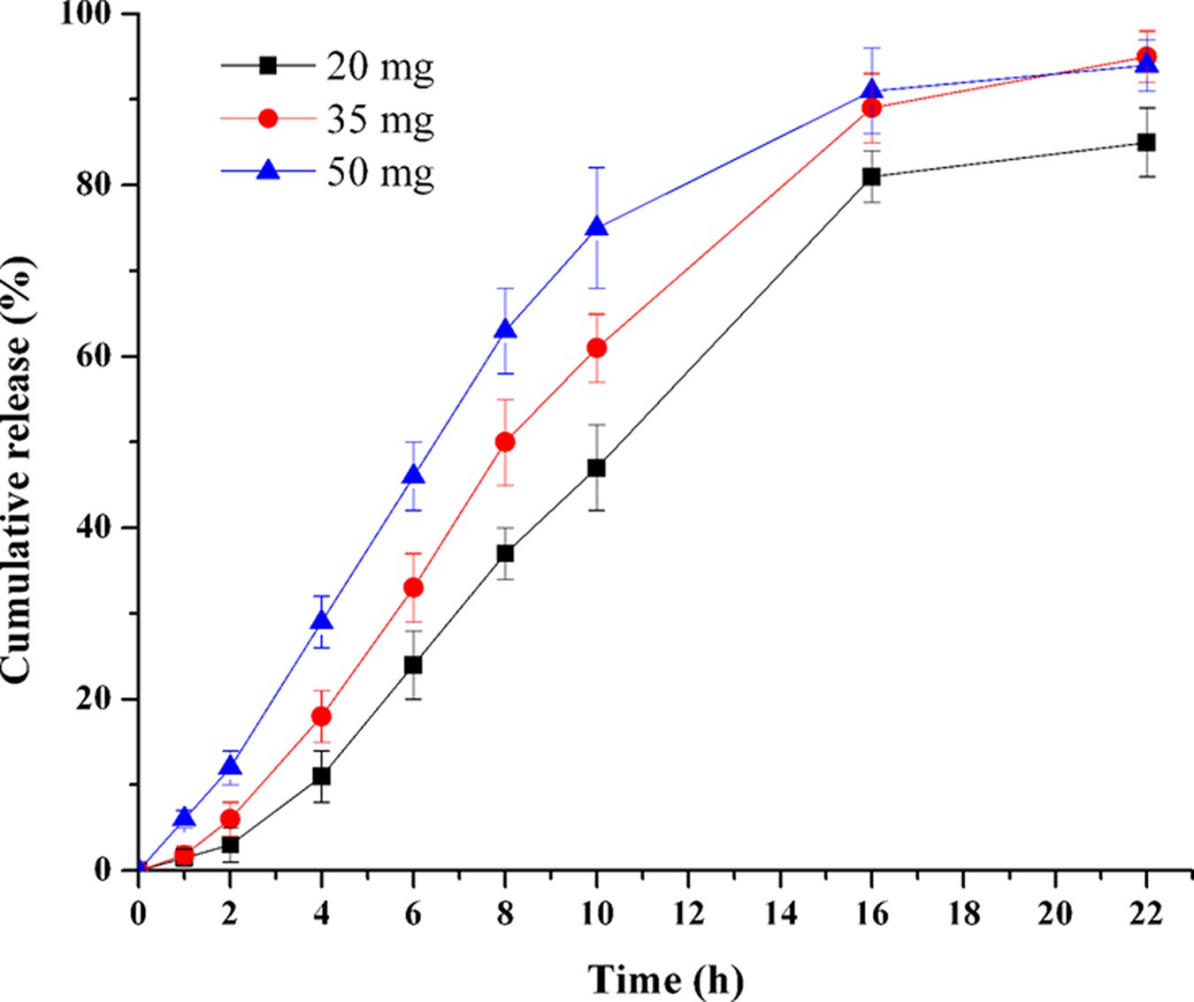
Effect of NaCl amount in push layer on TPM release (n = 6).

To study the influence of NaCl amount in push layer on drug release, tablet cores with various amounts of NaCl were prepared (F19-F21). As is showed in Fig 8, the TPM release rate increased significantly as the increase of NaCl from 20 to 50 mg. The *f*_2_ of formulations 20–35 mg, 35–50 mg and 20–50 mg was 48.7, 46.9 and 33.0, respectively. These *f*_2_ values of F19-F21 were all smaller than 50, which meant their release profiles were significantly different from each other. This demonstrated that NaCl amount in push layer was an important factor, as it could influence the TPM release significantly.

As is well know that osmotic agent could accelerate both the hydration and swelling rate of push layer [13]. The quicker the push layer hydrated and swelled, the greater the pushing force generated in the coating membrane and consequently rapider drug release. In some extreme cases, the pressure difference between the tablet inner and outside environments exceeded the limit of the membrane tension, the membrane would rupture.

### Influence of PEG in coating membrane

**Fig 9:**
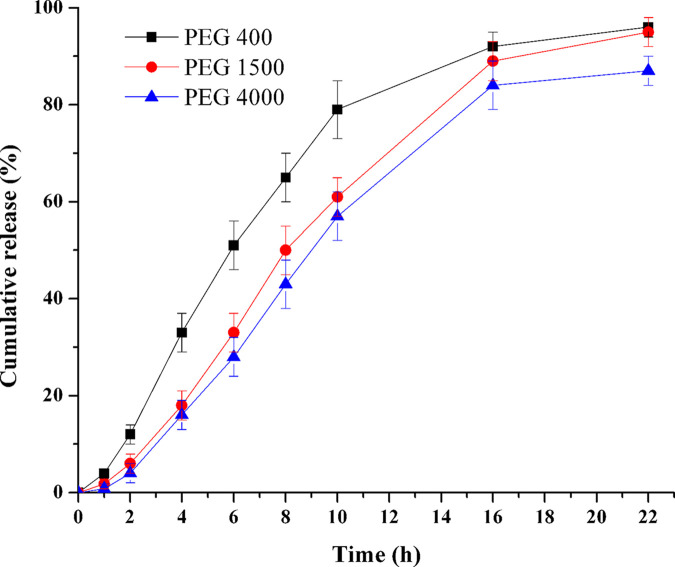
Effect of PEG molecular weight in coating membrane on TPM release (n = 6).

**Fig 10:**
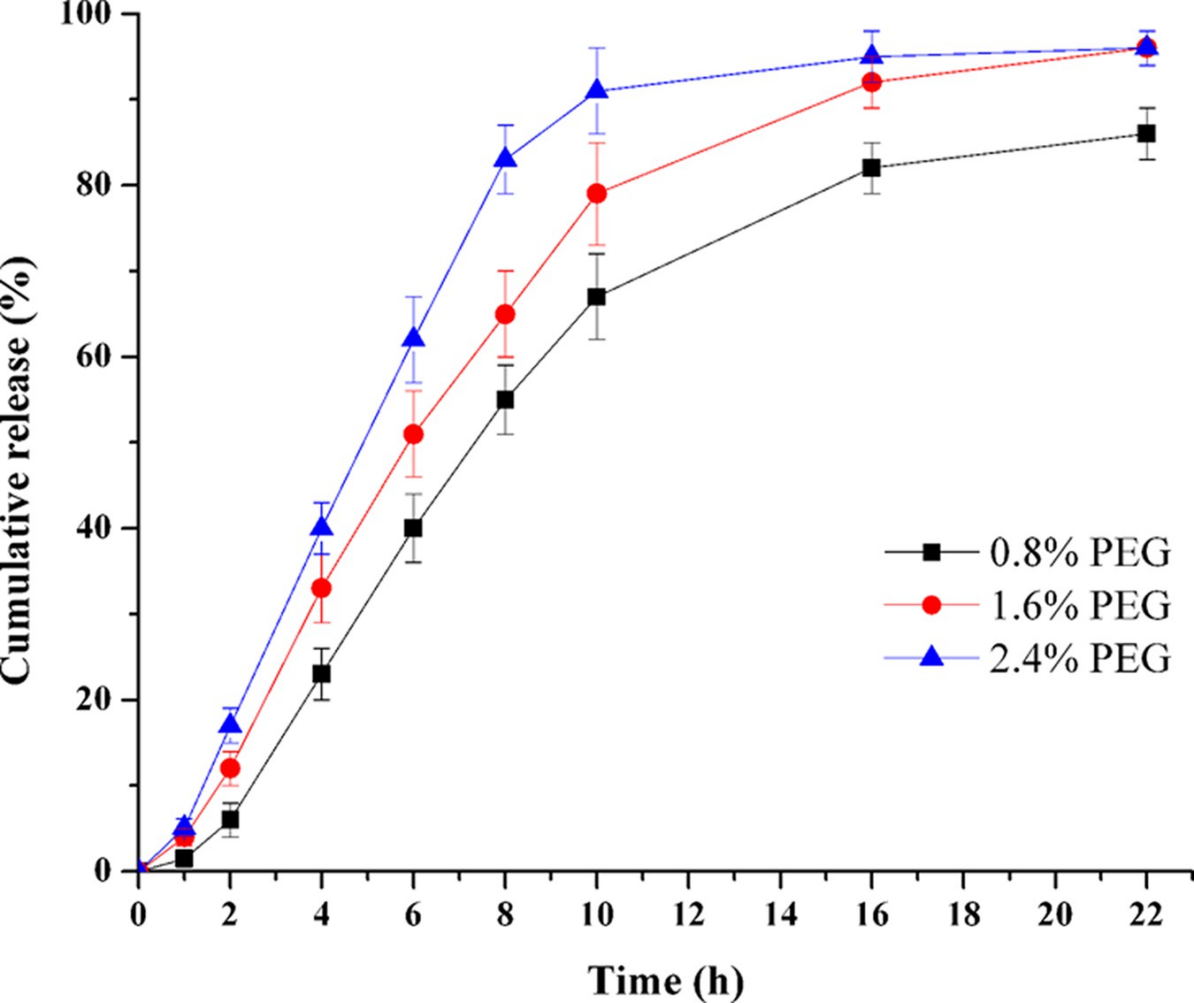
Effect of PEG level in coating membrane on TPM release (n = 6).

The membrane composition is a key parameter controlled the drug release rate. The tablet cores were coated by coating solution containing various types of PEG (F22-F24), and the influence of PEG Mw in the membrane was investigated. The results in Fig 9 demonstrated that the TPM release rate speeded up as the Mw of PEG decreased (*f*_2_: PEG 400–1500: 43.0; PEG 1500–4000: 66.7; PEG 400–4000: 37.4). Since PEGs in the three formulations (F22-F24) has the same weights, the lower Mw PEG would have relatively higher molecular numbers. Thereafter, the lower Mw PEG would cause more holes on the coating membrane and led to a faster drug release. Among the three categories PEGs, PEG 400 had the fastest drug release. To approach the ideal complete release time (8 h), PEG 400 with various amounts (0.8%, 1.6% and 2.4%, F25-F27) in the coating solutions were selected for further investigation. As exhibited in Fig 10, there was a correlation between the PEG level and release rate: the higher amount of PEG was, the rapider release rate would be (*f*_2_: 0.8%-1.6%: 49.8; 1.6%-2.4%: 48.7; 0.8%-2.4%: 34.8). It indicated that the PEG level in coating solution was a key factor influenced the drug release.

PEG was reported as a hydrophilic plasticizer, it could be leached easily and left behind porous structure, and thus enhanced the membrane permeability and drug release rate [30]. As the proportion of PEG increased in the membrane, more of PEG would dissolve in water. Hence, the membrane permeability increased and consequently increased the drug release rate [31].

### Influence of weight gain of tablet core

**Fig 11:**
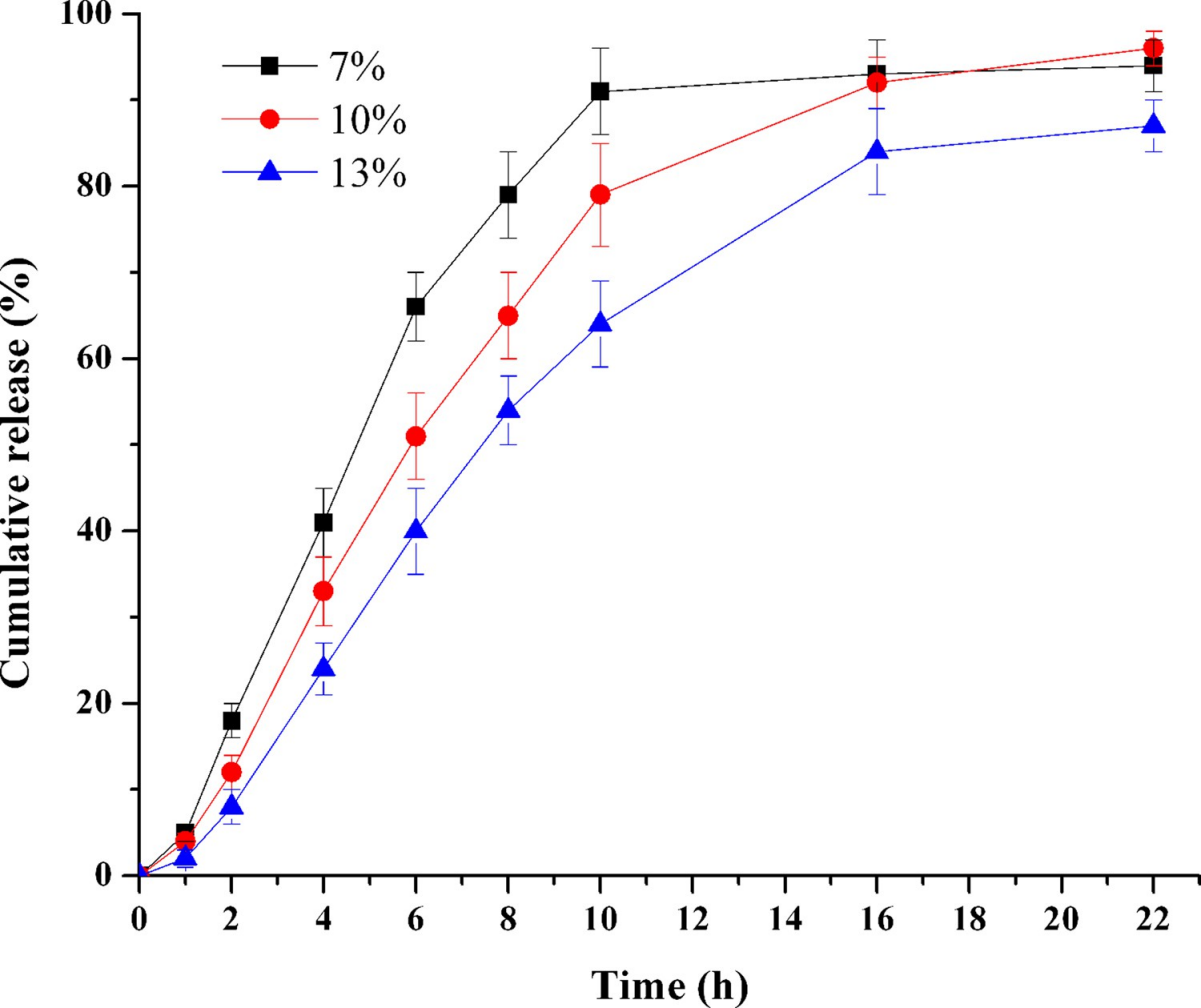
Effect of core tablet weight gain on TPM release (n = 6).

To study the influence of coating weight gain on drug release profile, tablet cores after coating with the weight gains of 7%, 10% and 13% were prepared (F28-F30), respectively. Fig 11 showed that the drug release rate decreased as the coating weight gain increased (*f*_2_: 7%-10%: 48.8; 10%-13%: 49.4; 7%-13%: 34.5). The thickness increase led to an increase of membrane resistance to water penetration, and resulting in a decreased drug release rate [32]. These results revealed that the weight gain of tablet core would influence the drug release significantly.

Film thickness is a function of the weight gain of tablets. As the weight gain of tablets decreased, the coating membranes would become thinner and resulted in weaker films. It should be noticed that, when the weight gain of tablet was too smaller, the pressure in the tablet might exceed the tensile strength of the membrane and cracked it during the period of drug release [27].

### Influence of orifice size

**Fig 12:**
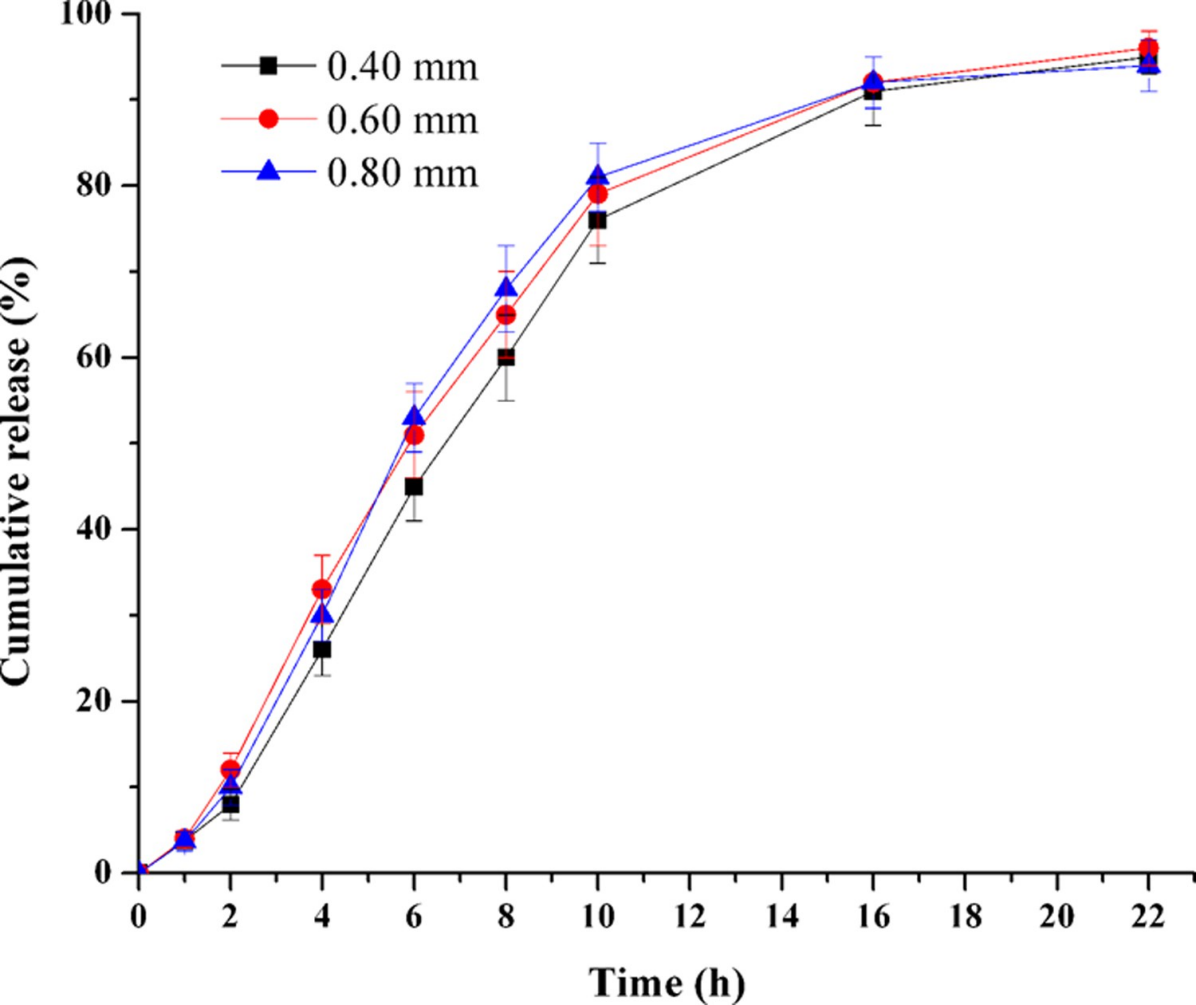
Effect of orifice size on TPM release (n = 6).

When the tablet core and membrane formulation were determined, the TPM release rate of the PPOP would be affected by the orifice size. The tablet cores were coated with the coating membrane (F29), and drilled on drug layer side surface with a round orifice at different diameters. As depicted in Fig 12, there was no significant difference among the release profiles of the tablets with orifice diameters ranging from 0.40 to 0.80 mm (*f*_2_: 0.40–0.60 mm: 65.6, 0.60–0.80 mm: 80.6, 0.40–0.80 mm: 63.0). For the 0.4 mm orifice PPOPs, the coating membrane of a few tablets would deform occasionally during the *in vitro* release tests, but this phenomenon did not happen in the 0.60 and 0.80 mm PPOPs. Thus, in the following studies the orifice diameter was fixed at 0.60 mm.

This result was consisted with the similar conclusion drawn by Liu et al. [19] and Xu et al [27]. The result was also accordant with that of EOP. It is reported that there is an appropriate range of orifice size for EOP. It should smaller than the maximum limit, to minimize the contribution to the delivery rate made by diffusion through the orifice; and it should larger than a minimum limit, to minimize the influence of hydrostatic pressure inside the system [33]. In the following studies, an orifice diameter of 0.60 mm within the appropriate range would be used continually.

### Optimization of the formulation

Based on the above exploration of single-factor experiments, the main factors for optimization were set as follows: (A) PEO amount in push layer; (B) NaCl amount in drug layer; (C) PEG amount in coating solution; (D) coating membrane weight gain of tablet cores.

The key factors were varied at four different levels. Osmotic pump tablets with various formulations were prepared according to L_16_ (4^5^) orthogonal design (Table 3). Similarity factor (*f*_2_) was employed to evaluate the release profiles of various formulations compared with the ideal release profiles [11]. For an ideal TPM-PPOP, the cumulative release percentage was assumed to be 0% at 0 h, and the ideal cumulative release percentage was supposed to be 90% at 8 h. Therefore, the equation of ideal release profiles was F = 11.25t, where F was the ideal cumulative release percentage at time t.

**Table 3:** Experimental plan and results of orthogonal design (L_16_ (4^5^)).

Run	Factor A	Factor B	Factor C	Factor D	*f*_2_
1	100	20	1.0	6	30.4
2	100	30	1.5	8	64.9
3	100	40	2.0	10	35.4
4	100	50	2.5	12	37.2
5	120	20	1.5	10	33.7
6	120	30	1.0	12	40.2
7	120	40	2.5	6	45.6
8	120	50	2.0	8	32.4
9	140	20	2.0	12	50.8
10	140	30	2.5	10	50.1
11	140	40	1.0	8	40.6
12	140	50	1.5	6	34.5
13	160	20	2.5	8	45.3
14	160	30	2.0	6	47.1
15	160	40	1.5	12	38.9
16	160	50	1.0	10	41.2
k1	41.975	40.050	38.100	39.625	
k2	37.975	50.575	43.225	45.800	
k3	44.225	40.125	41.425	40.100	
k4	43.125	36.550	44.550	41.775	
R	6.250	14.025	6.450	6.175	

k1, k2, k3 and k4 are the average sum scores of Level 1, Level 2, Level 3 and Level 4 for each factor; R is the range among the average sum scores of Level 1, Level 2, Level 3 and Level 4 for each factor.

The results of orthogonal design were showed in Table 3. The higher the *f*_2_ value was, the closer to the ideal release profile was. The level which got the highest *f*_2_ value was chosen as the optimal level of each factor. Therefore, A_3_B_2_C_4_D_2_ was selected to be the optimal formulation and it was found to be as follows: 140 mg PEO and 30 mg NaCl in push layer, 2.5% PEG in coating solution; 8% coating membrane weight gain of tablet cores.

### Evaluation of optimal formulation

**Fig 13:**
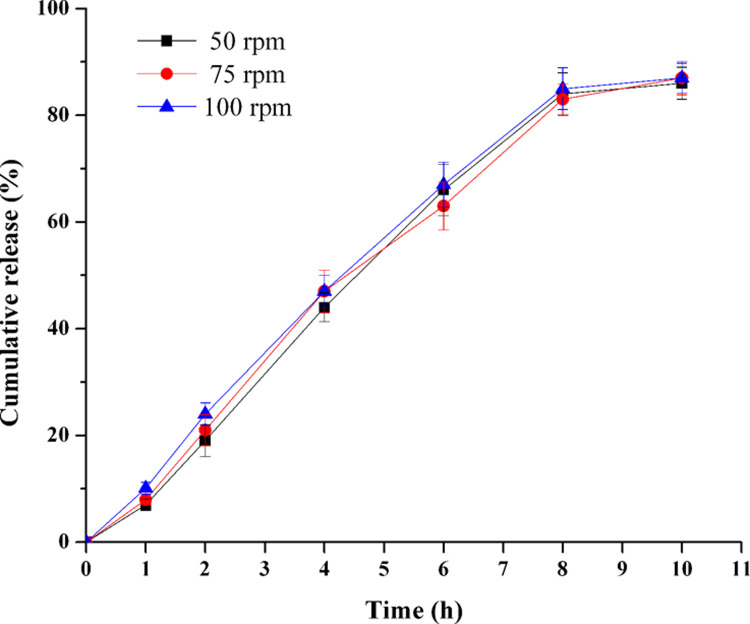
Effect of agitation rate on TPM release in optimal formulation (n = 6).

The push-pull osmotic pump tablets with the optimal formulation were prepared and *in vitro* release was carried out (Fig 13). Compared with the ideal release profile, the *f*_2_ of optimal formulation was 70.7, which was larger than 50 and implied that there was no statistical difference between the two profiles. To study the release kinetics, data of *in vitro* release test were simulated with zero-order (F = 10.92t – 10.575, R^2^ = 0.996), first-order (Ln(100—F) = -0.226t + 4.767, R^2^ = 0.957) and Higuchi (F = 31.01t1/2–13.43, R^2^ = 0.903) models, respectively [34]. As a result, the best fit was given by the zero-order equation due to the highest correlation coefficient, which suggested that the optimal osmotic pump tablet was able to deliver TPM at an approximate zero-order up to 8 h.

Dissolution tests were carried out at stirring rates of 50, 75 and 100 rpm [26] to investigate the effect of stirring rate on drug release profiles (Fig 13). It was found that the increase rate of stirring did not influence the TPM release profile from PPOP (*f*_2_: 50–75 rpm: 80.9; 75–100 rpm: 77.8; 50–100 rpm: 74.8).

**Fig 14:**
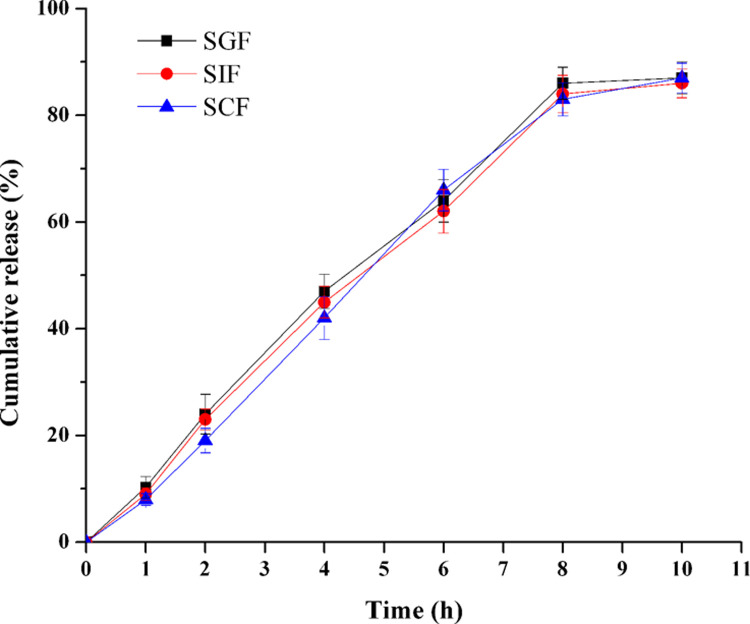
Effect of release media on TPM release in optimal formulation (n = 6). SGF: simulated gastric fluid; SIF: simulated intestinal fluid; SCF: simulated colonic fluid.

Furthermore, the release profiles of TPM-PPOPs were investigated in simulated gastric fluid (SGF), simulated intestinal fluid (SIF) and simulated colonic fluid (SCF), respectively [35]. The drug release profiles in these environmental media were showed in Fig 14. The results demonstrated that the gastrointestinal fluid scarcely affected TPM release, for the *f*_2_ among them were all larger than 50 (*f*_2_: SGF-SIF: 85.1; SIF-SCF: 75.4; SGF-SCF: 70.9).

As the drug release was independent of both agitation rate and release media, it may be speculated that the PPOP would exhibit a comparable *in vitro* / *in vivo* release profile [36].

### In vivo pharmacokinetic study

**Fig 15:**
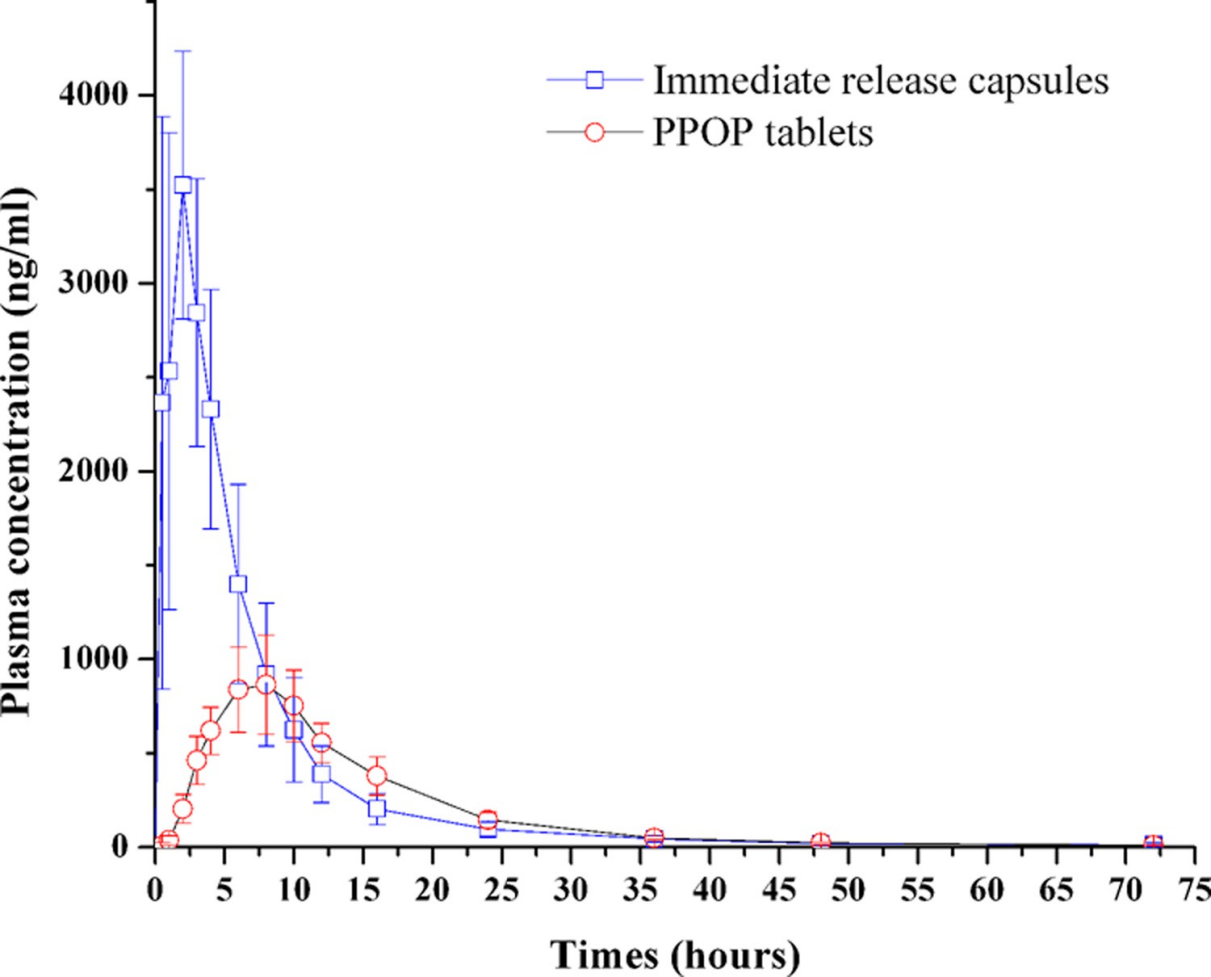
Mean TPM plasma concentration versus time after single dose (n = 6).

Plasma concentration versus time profiles of TPM after single dose, oral administration of TPM-PPOPs and immediate release capsules in beagle dogs were shown in Fig 15. As shown in Table 4, the t_max_ of PPOP tablets (6.69 ± 1.65 h) was remarkably longer than that of immediate release capsules (1.44 ± 0.68 h). Also, the mean residence time (MRT) of PPOP (13.72 ± 1.61 h) was significantly longer than that of immediate release capsules (6.98 ± 1.39 h). These results confirmed the prolonged release profile of PPOP formulations. In addition, the mean C_max_ of PPOP (893.58 ± 237.54 ng/mL) was remarkably lower compared with immediate release capsules (4368.75 ± 72.92 ng/mL). Apparently, the fluctuation of plasma concentration for PPOP was smaller than normal capsules. Therefore, PPOP may be desirable to provide a relatively stable plasma profile throughout the day and reduced the risks of severe adverse effects [10].

**Table 4:** Pharmacokinetic parameters of topiramate (TPM) in beagle dogs after a single dose oral administration of TPM push-pull osmotic pump (PPOP) tablets and immediate release capsules (n = 6).

Parameter	PPOP tablets	Immediate release capsules
*C*_max_ (ng/mL)	893.58 ± 237.54 *	4368.75 ± 72.92
*t*_max_ (h)	6.69 ±1.65*	1.44 ± 0.68
*t*_1/2_ (h)	9.29 ± 2.08	10.31 ± 2.43
AUC_0-t_ (ng·h/mL)	12765.53 ± 2622.91*	22645.83 ±5522.92
AUC_0-∞_ (ng·h/mL)	13070.25 ± 2693.75*	23164.58 ± 5550.14
MRT_0-t_ (h)	13.72 ± 1.61*	6.98 ± 1.39
*F*_*r*_ (%)	56.42 ± 11.46	—

* *p* < 0.05, compared with the immediate release capsules.

It was reported the effective blood concentration of TPM in human with status epilepticus was 2–40 μg/mL [37]. Based on this, the blood drug concentration of PPOP tablets here might be unable to produce sufficient therapeutic effect *in vivo*. This could be attributed to the incomplete drug release in dogs’ gastrointestinal tract. If the insufficient blood drug concentration of PPOP tablets in human beings was confirmed, the release pattern (or tablet dose) of prepared osmotic pump tablet needed to be adjusted. The concentration of TPM in plasma was almost zero after 36 h, while the elimination phase extended to 72 h, which could affect the parameters related about drug elimination greatly. Therefore, the parameters of elimination half-life (~10 h) was longer the references reported (3.7–5 h) [37]. It was reported that the mean plasma elimination half-life was 19–23 h in healthy volunteers without enzyme induction. When TPM was co-administered with enzyme-inducing antiepileptic drugs, plasma elimination half-life was reduced to 12–15 h in humans [38]. The elimination half-life was 0.5–1 versus 3.7–5 h in dogs with and without phenobarbital, respectively [37]. Caldwell et al. reported that 82% of topiramate was excreted unchanged in the urine in humans, while only 28% is excreted unchanged in dogs [39]. Therefore, TPM had a shorter elimination half-life in dogs than that in humans. The area under the plasma concentration-time curve (AUC) of PPOP tablets (13070.25 ± 2693.75) was significantly lower than that of immediate release capsules (23164.58 ± 5550.14). Compared with immediate release capsules, the relative bioavailability of PPOP tablets was around 56.4%. Similar phenomenon was reported by Gong et al. [12]. It could be explained like this: The length and transit time of dogs gastrointestinal tract were shorter than those of human beings, and the rigid structure osmotic pump tablet [11] would have less time to complete its drug release in the gastrointestinal tract, so when the PPOP tablet excreted from body, there was still a fraction of drug residual in the tablet, and thus resulted in the low AUC [12]. For the physiological difference of the gastrointestinal tracts between dogs and humans, drug absorption was likely to be more variable and less complete in dogs [40]. However, the actual reason for the lower relative bioavailability of TPM-PPOPs still need further study.

## Conclusions

The push-pull osmotic pump tablets of TPM had been successfully prepared by coating the bi-layer tablet cores. The optimal osmotic pump tablet was able to delivery TPM approximately in a zero-order pattern up to 8 h, which was independent of agitation rate and release media. *In vivo* study showed that both the t_max_ and MRT of TPM-PPOP were remarkably longer than those of immediate release capsules. These results confirmed the sustained release profile of TPM from PPOP formulation. Besides, drug plasma levels with lower fluctuations could also be achieved with TPM-PPOPs. This study showed that it was possible to formulate a sparely water-soluble drug as TPM into PPOP to prolong its release time and enhance its clinical safety.

## Supporting information

S1 Data (XLSX)
